# The impact of patient-reported outcome measures in clinical practice for pain: a systematic review

**DOI:** 10.1007/s11136-016-1449-5

**Published:** 2016-11-04

**Authors:** Michelle M. Holmes, George Lewith, David Newell, Jonathan Field, Felicity L. Bishop

**Affiliations:** 1 School of Psychology, University of Southampton, Southampton, Hampshire SO17 1BJ UK; 2 Primary Medical Care, University of Southampton, Southampton, Hampshire SO16 5ST UK; 3 Anglo-European College of Chiropractic, Bournemouth, Dorset BH5 2DF UK; 4 Back2Health, Petersfield, Hampshire GU32 3EH UK

**Keywords:** Patient-reported outcome measures, PROMs, Clinical practice, Pain

## Abstract

**Purpose:**

Patient-reported outcome measures (PROMs) have increasingly been incorporated into clinical practice. Research suggests that PROMs could be viewed as active components of complex interventions and may affect the process and outcome of care. This systematic review examines PROMs in the context of treatment for non-malignant pain.

**Methods:**

An electronic search on: MEDLINE, EMBASE, PsycINFO, PsycARTICLES, Cochrane Library and Web of Science identified relevant papers (February 2015). The inclusion criteria were: focused on implementing PROMs into clinical practice, adults, and primary data studies. Critical interpretive synthesis was used to synthesise qualitative and quantitative findings into a theoretical argument.

**Results:**

Thirteen eligible studies were identified. Synthesis suggested that PROMs may be included in the initial consultation to assess patients and for shared decision-making regarding patient care. During the course of treatment, PROMs can be used to track progress, evaluate treatment, and change the course of care; using PROMs may also influence the therapeutic relationship. Post-treatment, using PROMs might directly influence other outcomes such as pain and patient satisfaction. However, although studies have investigated these areas, evidence is weak and inconclusive.

**Conclusion:**

Due to the poor quality, lack of generalisability and heterogeneity of these studies, it is not possible to provide a comprehensive understanding of how PROMs may impact clinical treatment of non-malignant pain. The literature suggests that PROMs enable pain assessment, decision-making, the therapeutic relationship, evaluation of treatment and may influence outcomes. Further research is needed to provide better evidence as to whether PROMs do indeed have any effects on these domains.

## Introduction

Patient-reported outcome measures (PROMs) have increasingly been incorporated into routine clinical practice. PROMs are questionnaires collecting patient’s perceptions and views about their health [1–4]. These subjective evaluations are self-completed and typically produce numerical scores [5–7]. They are often used to assess pain, an inherently subjective and multifactorial construct which cannot be objectively measured [8, 9] and may be influenced by various factors including gender, age, and other socio-demographic characteristics. PROMs allow clinicians to capture patient views, feelings, and subjective experiences unlike traditional methods such as biophysical measures [10]. As PROMs are subjective, they inherently incorporate patients’ socio-demographic characteristics and background when measuring pain. PROMs can measure both health at a single point in time or long-term changes [5, 10, 11]. Standardised PROMs are validated to ensure: certainty over changes in scores, they can detect changes over time, and they measure the constructs they claim [4]. Additionally, PROMs can be used for audit to examine service effectiveness, appropriateness, quality, and performance [7, 12].

In the early 1990s, PROMs had three main uses within clinical practice: increase knowledge concerning disease trajectories, evaluate effectiveness of treatment on individual patients, and assess the quality of care provided [13]. These outcomes were suggested to be intrinsically linked to processes of providing quality health care [13, 14]. As part of moves to value patients’ views in health care, PROMs have been routinely collected during four procedures in the UK National Health Service (NHS) since April 2009: unilateral hip replacements, unilateral knee replacements, groin hernia surgery, and varicose vein surgery [5].

Greenhalgh and Meadows [15] provided one of the first reviews to identify how PROMs might improve health care. They aimed to assess the current evidence base. Their review aimed to assess the current evidence base surrounding the use of PROMs in routine clinical practice by examining randomised controlled trials (RCTs) exploring this topic. The authors found limited evidence that PROMs may influence the detection of psychological problems and facilitate communication between clinicians and patients [15].

A number of other reviews have since been conducted assessing the impact of using PROMs in clinical practice, examining evidence from RCTs or controlled trials. To address claims that PROMs could provide additional information to clinicians and improve patient care, Espallargues et al. [16] systematically assessed the effectiveness of providing feedback on PROMs to clinicians. They concluded that the impact of providing feedback on PROMs to clinicians was unclear, but using PROMs may modify elements of healthcare provision through increased detection and diagnosis of conditions and subsequent service utilisation [16].

Boyce et al. [17] examined qualitative research on clinicians’ experiences of using PROMs. Some clinicians viewed PROMs to potentially impact on care, by influencing communication, shared decision-making and planning treatment [17].

Whilst these reviews provide interesting insights into the potential impact of PROMs on clinical outcomes when used in clinical practice, each review focused on either trials or qualitative literature. Cullum and Dumville [18] argue that to understand complex interventions, all relevant studies using a broad range of designs must be identified and synthesised.

Research to date argues that PROMs may be viewed as active components of clinical interventions, potentially affecting process and outcomes of care. However, studies on PROMs in non-malignant pain have not been reviewed. Therefore, we conducted a systematic review of the literature on implementing PROMs in clinical practice in non-malignant pain. Previous reviews indicate that PROMs may have complex effects on care with a variety of outcomes [1, 16, 19].

As no previously published reviews examine PROMs in the context of non-malignant pain and previous literature on generic use of PROMs has shown mixed benefits, there were no hypotheses set out at the start of the review. The review aimed to identify all relevant evidence and examine any emerging concepts from published findings as a first investigation of the potential impact(s) of implementing PROMs in routine clinical practice on the process and outcome of health care for non-malignant pain. Based on previous reviews, it was suggested the impact of PROMs in non-malignant pain may include elements of the patient–clinician encounter, process of care, patient behaviour, as well as outcomes of health care. The review was not limited to these areas and included impacts demonstrated in trials but also those suggested by qualitative and survey studies, based on patients’ and clinicians’ experiences.

## Methods

### Review methodology

Previous reviews examining PROMs in clinical practice have found studies to be heterogeneous [1, 16, 19] finding meta-analysis to be unjustified; therefore, meta-analysis was deemed inappropriate for this review [20]. This review used critical interpretive synthesis (CIS), a method of synthesis developed from meta-ethnography. CIS was developed as an alternative to traditional quantitative systematic reviews or qualitative syntheses, because researchers and healthcare professionals must examine diverse bodies of evidence to resolve complex problems within health care. CIS was thus designed to use both qualitative and quantitative literature to assemble arguments from all the available evidence, despite varying study designs [21, 22]. Synthesising the results of qualitative and quantitative research improves the understanding of a complex phenomenon by viewing it from multiple perspectives; trials can identify the effectiveness of an intervention in a certain context, with qualitative studies and surveys further exploring the potential impact of an intervention through participants’ views and lived experiences [23]. CIS also includes papers of high and low methodological quality, as all may have at least some relevance, although this is accounted for in the synthesis process [21]. The interpretive stages of CIS (outlined below) permit theoretical concepts from a diverse body of literature to be combined in order to generate a richer understanding of the phenomenon of interest.

### Search strategy

This review followed established guidance regarding search strategies, inclusion and exclusion criteria, and data extraction [24, 25]. CIS guidance suggests literature searches should be broad and flexible and multiple methods were used to obtain relevant studies [21]. Several relevant databases were searched in January 2015: Medical Literature Analysis and Retrieval System (MEDLINE); Excerpta Medical Database and Allied and Alternative Medicine (EMBASE); PsycINFO; Cochrane Library; Web of Science; and PsycARTICLES. Terms included derivatives of patient-reported outcomes and clinical practice (see Table 1). The search was restricted to items published after 1985, when PROMs emerged in the literature [26]. Additional searches were conducted on: Google Scholar, the UK Clinical Research Network Study Portfolio website, bibliographies on obtained studies, and key author publications.

**Table 1:** Example search strategy

Patient outcome assessment [thesaurus term] OR process assessment (health care) [thesaurus term] OR outcome assessment (health care) [thesaurus term] OR “patient-reported outcome*” [keyword] OR self-report [thesaurus term] OR self-assessment [thesaurus term] [thesaurus term]
AND
“clinical practice” [keyword] OR “clinical setting” [keyword] OR “practice setting” [keyword]

**Fig. 1:**
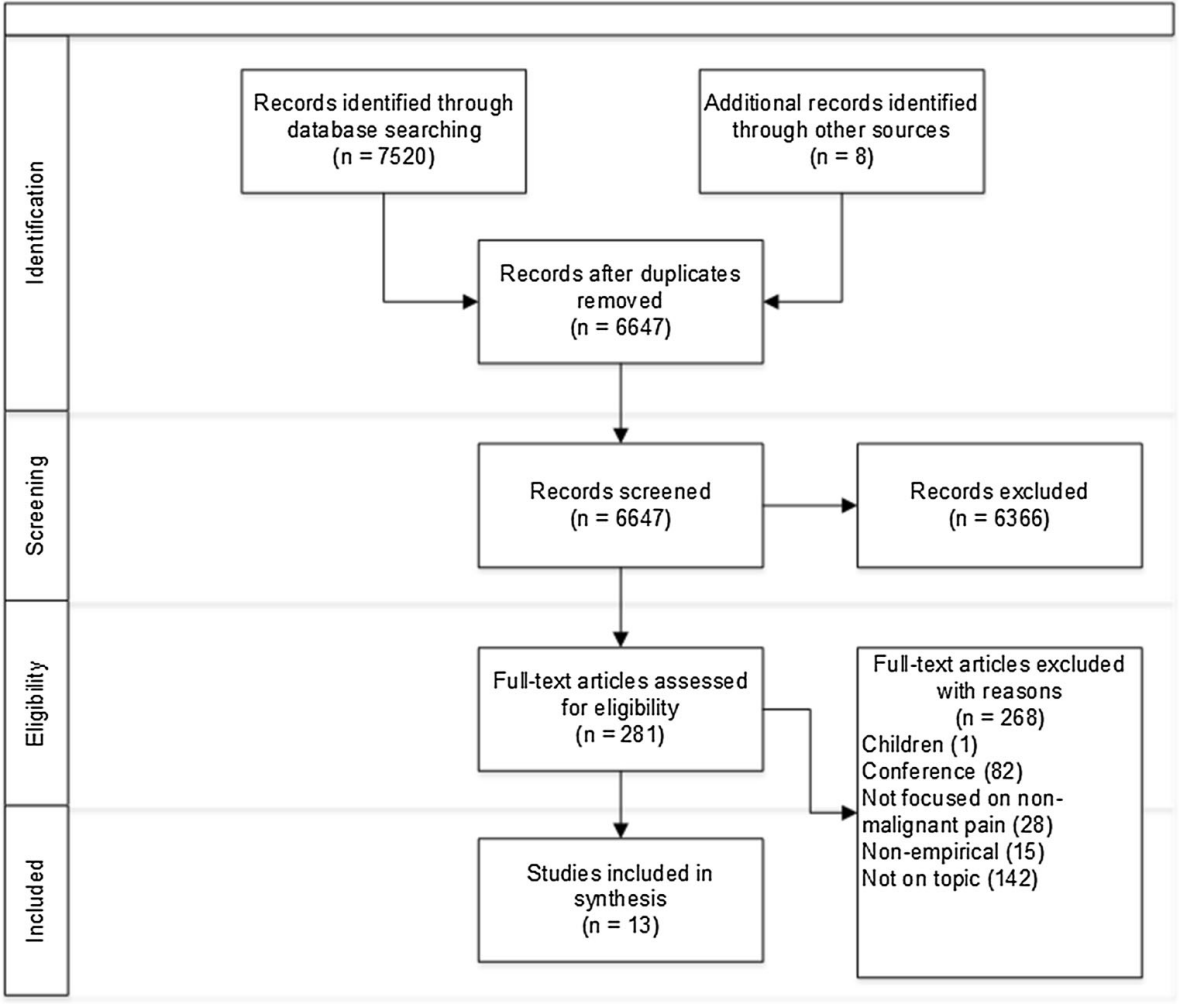
Prisma flowchart

Study selection was predetermined by inclusion and exclusion criteria (see Table 2). During the screening process, one article was translated from Portuguese to determine eligibility. Full texts were examined and a list of potential studies generated by one reviewer, before two reviewers finalised studies for inclusion. See PRISMA flowchart in Fig. 1 [27]. The initial search was very broad, and it generated a lot of irrelevant studies; however, CIS encourages a broad and inclusive approach. The aim was not to obtain a representative sample but to obtain a comprehensive sample of all papers that met the inclusion criteria.

**Table 2:** Inclusion/exclusion criteria

Inclusion	Exclusion	Justification of criteria
Study objectives included to: explore, examine, evaluate, demonstrate, assess the impact of implementing PROMs into routine clinical practice	No objectives to explore or examine the impact of implementing PROMs into routine clinical practice	Studies were restricted to those exploring PROMs use in clinical practice, excluding studies investigating their use in research. Studies which evaluated the use of PROMs as part of a larger intervention, such as counselling, were also not included as the results may not be specific to the PROMs intervention
Adult patients (aged ≥ 18) with non-malignant pain or within healthcare settings which specifically see patients with non-malignant pain	Adult patients without pain, patients with malignant pain, general healthcare settings (such as outpatients, emergency clinics, general practice patients and specialist services) where the patients may not have pain. Children or adolescents (<18)	These restrictions were placed as the experiences and treatment of malignant pain may be different to those with non-malignant pain. Children were also excluded due to the biological and psychological differences between children and adults
Primary studies (quantitative studies; qualitative studies; mixed-method studies)	Letters; conference abstracts; editorials, commentaries; reviews; dissertations; books	Studies were restricted to empirical literature, to examine the potential impact of PROMs rather than theoretical concepts of their use

### Data synthesis and assessment of confidence

This review used CIS to synthesise the emerging concepts underlying the potential impact of using PROMs in clinical practice. Table 3 depicts the stages of synthesis. CIS mapped the qualitative literature against the quantitative to balance the inherent limitations of each method and provide further explanations of the results (for example of mapping see Table 4). Data interpretation by three reviewers refined constructs.

**Table 3:** Stages of synthesis

Stage One	Detailed inspection of papers, documented with descriptive synthesis of studies and data tabulation of study characteristics
Stage Two	Refining of results through translation; translation occurs through detailed extraction of study results, followed by grouping and clustering of the results
Stage Three	Synthesising findings using Reciprocal Translation Analysis (RTA). RTA uses frameworks to compare the results of each study and interpret all the evidence
Stage Four	Concept mapping was used to integrate the evidence into a single framework called a synthesised argument. The synthesised argument aims to explain the synthetic constructs produced in step 3 and the relationship between studies in order to answer the overarching research questions

**Table 4:** Construct mapping example

Sub-construct: referrals	Positive effect	Adverse or no effect
*Quantitative*	33% of clinicians felt that health status reports contributed to patient referrals some of the time [39]	50% of clinicians felt that health status reports did not contribute to making patient referrals [39]
		Non-significant difference in additional treatment post-implementation of a numerical rating scale (*p* = .461) [43]
	17% of clinicians felt that health status reports contributed to patient referrals most of the time [39]	
		Reducing doctor visits was found to be non-significant after the use of PROMs [39]
		Arthritis-related referrals was found to be non-significant after the use of PROMs [39]
*Qualitative*	Based on the scores, clinicians chose to refer the patient to another service [34]	

The CERQual tool was used to assess confidence in evidence for each of the concepts generated during the synthesis [28]. CERQual helps reviewers judge if the concepts are representative of the phenomenon being studied. The CERQual has four components which contribute to assessing the confidence of each review finding: methodological limitations, relevance, coherence, and adequacy of data (see Table 6). Examining the components, an overall assessment was made on the confidence on the concepts [28]. A final table was then developed summarising the concepts from the synthesis and the CERQual assessments. This assessment of confidence fits with the principles of CIS, which assembles arguments from all available evidence despite varying study designs and methodological quality. By using the CERQual assessment, we were able to formally assess confidence in the assembled constructs and overall synthesised arguments.

To examine methodological quality and risk of bias of the primary studies [29], questions were extracted from the Mixed-Method Appraisal Tool (MMAT) [30]. The MMAT provided a single method of analysing methodological quality for all studies, rather than applying various checklists to different studies [30]. The MMAT allows for studies to be assessed according to study design, and each is then evaluated on four criteria [30]. For example, randomised controlled trials are assessed on their randomisation, blinding, outcome data, and drop-out, with qualitative research being assessed on sources of data, analysis, context, and researchers’ influence. Other tools were examined for relevance to the review, but were deemed inappropriate due to the heterogeneity of the results, not allowing for assessment of the quality of the research in respect to the study design [31, 32]. This assessment provided an overview of study quality and methodological implications of the study, which was used when synthesising the study results. The two MMAT screening questions were modified to include the five appraisal prompts used for judging study quality in CIS [21].

## Results

Thirteen eligible studies were identified (see Table 5); including: two qualitative studies, one mixed-method study, two RCTs, two non-randomised trials, two case series, one case–control study, two case series, one audit, one case report, and one cross-sectional analytic survey. The studies included patients and clinicians as participants. A variety of PROMs were used across the studies (see Table 5). PROMs were commonly completed on paper, with one study using computer software [33].

**Table 5:** Study characteristics

Authors	Country	Study design and method	Study aim	PROMs used (* validated measure) and concepts measured	Setting and participants	Analysis
Bottega et al. [37]	Brazil	Qualitative description; open-ended questionnaire	To explore nurses’ views on using a PROM to assess pain	Visual Analogue Scale—pain levels	Hospital. *n* = 14 nurses	Thematic analysis
Boyce et al. [17]	Ireland	Qualitative description; interviews	To explore surgeons’ experiences of using PROMs, to identify practical and methodological challenges, as well as identifying attitudes on the value of the feedback and the potential impact the information had on clinical practice.	Oxford Hip Score (OHS)*—hip pain and function, ED-5Q*—health status, Hip Osteoarthritis and Outcome Score (HOOS)* pain, symptoms, activity of daily living, sport and recreation function and hip-related quality of life	Primary hip replacement surgery. *n* = 11 Surgeons	Framework analysis
Buchi and Sensky [36]	Not known	Case series; patient-reported outcome measures	To demonstrate the application of PRISM in clinical practice and how it can be used facilitate patient-clinician communication	PRISM*—burden of suffering due to illness	General Hospital—Psychiatry. Two patients—1 female (33) multiple sclerosis; 1 male (58) severe multiple trauma	Quantitative descriptive
dos Santos Silva et al. [42]	Brazil	Non-randomised controlled trial; patients’ medical reports	To test the hypothesis that training for nurses of applying a systematised pain assessment of pain control effects decision-making regarding administration of morphine and affects pain relief for patients	Numeric Pain Rating Scale—pain level	Cardiac Surgery. *n* = 182 Cardiac surgery patients; mean age—55.7	Correlation of variables—Chi-square, Likelihood Ratio Test; Descriptive statistics; Comparison among groups—Kruskal–Wallis and Dunn test
Hadjistavropoulos et al. [44]	Canada	Non-randomised controlled trial; patient-reported outcome measures	To assess whether systematic pain assessment changes the clinical practice of medical practitioners	21-point box scale*—pain levels, Geriatric Pain Measure (GPM)*—pain intensity, Geriatric Depression Scale (GDS-SF)*—depression	General Practice. *n* = 114 seniors with complex medical problems; mean age–80.74	T tests
Hvitfeldt et al. [33]	USA/Sweden	Mixed-method—qualitative description and cross-sectional analytic study (triangulation design); questionnaires; Semi-structured interviews	To identify the properties of a patient-reported measurement system in two different contexts.	Oswestry Disability Index*—low back pain disability, SF-36*—health-related quality of life, Musculoskeletal Outcomes and Data Evaluation and Management System (MODEMS)—unknown	1 Spine Centre (USA); 2 Rheumatology Clinics (Sweden). *n* = 88 clinical patients; *n* = 18 healthcare providers (15 MDs, 2 physiotherapists, 2 nurse practitioners)	Quantitative data—Fisher’s exact 2-tailed test; qualitative data—Content analysis
Kazis et al. [39]	USA	Randomised controlled trial; questionnaires, patients’ medical records	To investigate the value of health status information on clinical practice for patients with rheumatoid arthritis.	Arthritis Impact Measurement Scales (AIMS)*—physical, social and emotional wellbeing, Modified Health Assessment Questionnaire (MHAQ)*—health status	Arthritis Centres. *n* = 1920 patients with rheumatoid arthritis. *n* = 24 physicians	Analysis of variance F-tests, if significant, pair-wise comparison using *t* tests
Mularski et al. [43]	USA	Case–Control; patients’ medical records	To measure the impact of using a PROM on the quality of pain management	Numeric Rating Scale—pain intensity	Veteran Affairs Medical Centre. *n* = 600 patients	Multivariate logistic regression.
Purser et al. [41]	UK	Audit; patients’ medical records	To assess whether use of pain assessment affects the pain management behaviour of nurses	Numeric Rating Scale—pain levels	General Hospital. Stage One, *n* = 202, Stage Two, *n* = 60, stage Three, *n* = 253 (medical and surgical patients)	Descriptive statistics
Ravaud et al. [45]	France	Cluster-randomised controlled trial; outcome measures, patients’ medical record	To evaluate the impact of an educational programme for nurses to improve pain assessment	Visual Analogue Scale—pain intensity	Surgical wards. *n* = 2278 surgical patients	Mixed-model ANOVA
Schorn et al. [35]	USA	Cross-sectional analytic; survey	To assess how well a tool for pain measurement is received by healthcare providers	PEG (3-item version of the Brief Pain Inventory)*—pain intensity and interferences, Patient Health Questionnaire (PHQ-4)*—depression and anxiety, Generalised Anxiety Disorder (GAD-7)*—anxiety	Primary care. *n* = 30 primary care providers	Quantitative data—descriptive statistics, qualitative data—content analysis
Stratford and Binkley [40]	Canada	Case series; patient-reported outcome measures	To demonstrate the application of the Roland-Morris questionnaire in clinical scenarios can aid decision-making in clinical practice	Roland-Morris Questionnaire*—disability	Physical therapy. *n* = 3, patients with low back pain	Quantitative descriptive
Thigpen and Shanley [34]	USA	Case report; patient-reported outcome measures	To demonstrate how PROMs can aid clinical practice in rehabilitation settings	Disabilities of Arm, Shoulder and Hand (DASH)*—upper extremity disability, DASH Sports Module (DASH-SM)—symptom and function, Pennsylvania Shoulder Score (PENN)*—pain, satisfaction and function, SF-12*—general health status	Physical therapy. *n* = 1, patient with shoulder pain	Quantitative descriptive

**Fig. 2:**
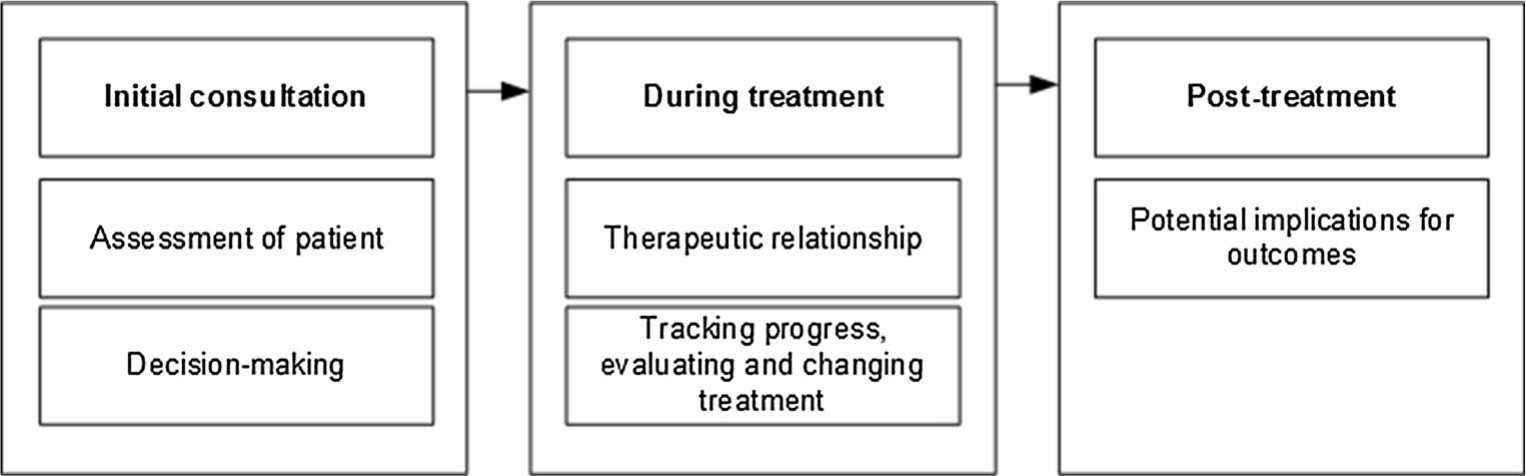
Concept map

Five synthetic constructs were developed using reciprocal translational analysis (RTA)—see Table 3. The five constructs are: assessment of patient, decision-making, therapeutic relationship, tracking progress and evaluating and changing treatment, and potential implications for outcomes. A concept map (Fig. 2) was created depicting the five key areas in which PROMs are suggested to impact clinical practice and relates these to three stages of treatment (initial consultation, during treatment, and post-treatment). Table 6 shows an assessment of the evidence supporting each construct.

**Table 6:** CERQual summary assessment

Review finding	Studies contributing to the review finding	Studies contradicting the review finding	Assessment of methodological limitations (the extent of problems in the design, conduct or analysis of primary studies contributing to the construct)	Assessment of relevance (the extent to which the primary studies contributing to the construct are applicable to the context of the review)	Coherence (the extent to which the construct is supported within the primary studies in the review)	Adequacy of data (determination over the detail from the primary studies contributing to the construct)	Confidence
Assessment of patient	Bottega and Fontana [37], Buchi and Sensky [36], Kazis et al. [39], Schorn et al. [35] and Thigpen and Shanley [34]	Boyce et al. [17], Kazis et al. [39]	Moderate methodological limitations	Indirect relevance	Minor concerns about coherence (data are reasonably consistent within and across all studies)	Minor concerns about adequacy (5 studies offering moderately rich data)	Moderate confidence
Decision-making	Bottega and Fontana [37], Boyce et al. [17], Buchi and Sensky [36], Hvidfeldt et al. [33], Stratford and Binkley [40], Thigpen and Shanley [34]		Moderate methodological limitations	Indirect relevance	No concerns about coherence (data consistent within and across all studies)	No concerns about adequacy (6 studies offering moderately rich data)	High confidence
Therapeutic relationship	Bottega and Fontana [37], Buchi and Sensky [36], Hvitfeldtet al. [33], Schorn et al. [35], Thigpen and Stanley [34]	Kazis et al. [39], Schorn et al. [35]	Moderate methodological limitations	Indirect relevance	Minor concerns about coherence (data are reasonably consistent within and across all studies)	Minor concerns about adequacy (5 studies offering moderately rich data)	Moderate confidence
Tracking progress, evaluating and changing treatment	Bottega and Fontana [37], Boyce et al. [17], dos Santos Silva et al. [42], Hvidfeldt et al. [33], Kazis et al. [39], Purser et al. [41], Schorn et al. [35], Stratford et al. [40], Thigpen and Stanley [34]	Boyce et al. [17], Hadjistavropoulos et al. [44], [39], Mularski et al. [43], Schorn et al. [35]	Moderate methodological limitations	Indirect relevance	Major concerns about coherence (data are not consistent within and across all studies)	Minor concerns about adequacy (7 studies offering moderately rich data, 2 studies offering thin data)	Low confidence
Potential implications for outcomes	dos Santos Silva et al. [42], Hadjistavropoulos et al. [44], Ravaud et al. [45]	dos Santos Silva et al. [42], Hadjistavropoulos et al. [44], [39], Ravaud et al. [45]	Moderate methodological limitations	Indirect relevance	Major concerns about coherence (data are not consistent within and across all studies)	Substantial concerns about adequacy (3 studies both offering thin data)	Very low confidence

### Assessment of patient

One prominent use of PROMs was to assess patients’ pain. Clinicians from various backgrounds, including physicians, nurse practitioners, and physical therapists, suggested that the purpose of PROMs was to assess the patients’ pain and quantify the impact of their pain [34, 35]. PROM data were also seen as a useful way to view pain within the context of a patients’ life [36, 37]; illustrated in the following quote from a nurse using PROMs in a hospital setting: *It is important to assess and take into account the thresholds of physical pain for each different individual on different occasions and how it is impacted by cultural and physiological factors* [37]. Collectively, the qualitative literature suggested that PROMs were thought to provide a positive method of gathering essential information from patients. However, there is little information on participant characteristics or recruitment for these studies, so this finding may not reflect the population of interest, patients with non-malignant pain.

In one qualitative study, orthopaedic surgeons raised concerns over PROM data, seeing the data as highly subjective and questioning the patients’ ability to provide “objective” data on their pain [38]. A quote from a surgeon provides a powerful illustration of this: *Getting patients to fill out forms is grossly inaccurate in my book… the patient 9 time(s) out of 10 wouldn’t understand what hip pain is* [38].

Kazis et al. [39] explored physicians’ views through a survey on the contribution of health status reports generated from PROMs. The majority of clinicians felt that PROMs impacted overall patient assessment in some or all of their consultations and the reports contributed to medical history taking. Thirty-eight per cent of clinicians also felt that the reports contributed to physical examination during some or most of their consultations. Other clinicians felt that no contribution was made to overall patient examination, medical history taking, or physical examination. However, not all of the clinicians surveyed had been sent the health status reports and used them in practice, although some of their patients had completed the PROMs as part of an RCT. Their lack of experience using PROMs may have significantly influenced their views on how PROMs contribute to patient assessment.

The outcomes related to this construct are inconclusive. Clinicians had mixed views when surveyed on whether PROMs may contribute to patient assessment. Similarly, in the qualitative studies clinicians suggested PROMs had benefits in this area but also voiced concerns about the validity of PROMs.

### Decision-making

Clinicians felt that PROMs made valuable contributions to the decision-making process surrounding care. Across three qualitative studies, clinicians including medical doctors, surgeons, and nurses claimed that PROMs facilitated shared decision-making [33, 37, 38]. This is demonstrated in a quote from a Swedish healthcare provider, after PROMs had been implemented into their clinic for two years: *Work is smoother, it is much easier to form an opinion and decisions are easier to make* [33]. PROMs were thought to provide useful information to choose an appropriate treatment for a patient and develop a treatment plan.

PROM scores also enabled clinicians to provide individualised treatments based on patients’ needs and direct them to appropriate care [34, 37]. Within a study on nurses’ use of PROMs, a nurse stated: *This method is of great value in the performance/assistant of planning so we can assign a more expressive care in relation to the pathology and the patient as a whole. Thus, seeking to minimise the patient’s suffering and pain* [37]. Using PROMs in decision-making enabled clinicians to feel they had enough information to develop individualised treatment plans.

PROMs were also used in the decision-making process to enable clinicians to set functional goals with patients. Two case series and a case report examined how PROMs were used for goal setting [34, 36, 40]. PROMs provided baseline data on patients’ current situation and then used to anticipate change and set goals.

No studies quantitatively tested the hypothesis that using PROMs improves shared decision-making. However, the qualitative literature does suggest that shared decision-making improves and decisions are increasingly individualised with PROMs.

### Therapeutic relationship

The synthesis suggested that PROMs had an impact on the therapeutic relationship between patients and clinicians through improving communication and patient engagement regarding their care.

A case report demonstrated how PROMs were used to improve communication between patient and physical therapists and start dialogue regarding their care [34]. Although the authors did not provide adequate details of the procedure and analysis, other studies demonstrated similar findings. For example, in one study both patients and clinicians believed that using PROMs changed the clinician–patient interaction, as this patient explained: *The system made it possible for the provider and I to talk about the important issues* [33]. In a survey of primary care providers (PCPs), all using PROMs in their clinical practice, 76% felt satisfied that the PROM measuring pain helped patients participate in their pain management [35].

Other qualitative findings also suggested that clinicians believed PROMs enabled patients to get involved in their care. This included identifying patient concerns and engaging patients in self-management [33, 35, 36]. One nurse stated: *I see the implementation of the pain scale as a way to humanize care, where we can stop relying on machines and turn to the patient; to what he is saying and feeling. Giving them an active voice and a right to express themselves* [37]. This humanisation of care, aided by communication and patient engagement, was thought to improve the relationship between patients and clinicians. Similarly, in a survey of doctors (some of whom had experienced PROMs and some of whom had not) the majority felt that PROMs contributed to the doctor–patient relationship, although the survey did not examine whether this contribution was positive or negative [39]. However, qualitative literature suggests that PROMs may facilitate interactions, aid communication, and promote individualised care. It is through these processes, that PROMs may improve the therapeutic relationship.

### Tracking progress and evaluating and changing treatment

Several studies demonstrated using PROMs for the tracking of patient progress, using the scores from PROMs to evaluate treatment and change treatment plans accordingly.

A survey found that 53.3% of PCPs were satisfied that the PROM helped them to understand patient progress [35]. A case series also suggested that information from PROMs was used to track progress [40]. Finally, this use of PROMs was also demonstrated by nurses: *This scale is important in the sense of monitoring the evolution of the intensification of pain and even to what point the treatment is being beneficial to the patient* [37].

Despite these findings, only 39.9% of PCPs felt satisfied that PROMs helped them to modify a treatment plan [35]. Several clinicians from two studies did not feel that the PROMs helped them modify a treatment plan [35, 38]. Several surgeons raised concerns over the information provided from PROMs, one surgeon stated: *I just think there is a lot of effort being put in there for not a lot of surgical gain from my perspective* [38]. Thirty per cent of PCPs surveyed on PROM use were dissatisfied regarding PROMs to help them to modify a treatment plan [35]. However, these surveys specified neither previous treatments nor future planned treatments.

Nonetheless, clinicians from several studies reported that PROM scores did influence treatment plans, and this was done on both an individual patient level and clinician level. A qualitative study on surgeons, PROMs encouraged two clinicians to reflect and change their clinical practice [38]. Individually patients’ treatments were also affected, one nurse stated: *It is (sic) tool that allows us to quantify the pain our patient is feeling with more accuracy, and rethink whether or not the therapy being given is really effective in treating that individual* [37].

As part of the construct on tracking progress and evaluating and changing treatment, two sub-constructs were generated: using PROMs to change patient medication use and using PROMs to change referrals to other clinicians and health services. One case report suggested PROM scores were used to refer the patient to another service [34]. Doctors surveyed on PROM use had conflicting opinions; 50% of doctors felt that health status reports (generated from PROM data) did not contribute to patient referrals, and 54% of doctors felt that reports did not impact on medication decisions [39]. However, not all doctors had used PROMs in practice.

Five studies tested the impact of PROMs on medication decisions. One study found that 17% of patients had analgesia altered and 6% of patients had an additional dose of analgesia after PROMs had been implemented across a hospital [41]. Another study, which issued nurses with training on PROMs and implemented PROMs across a cardiac surgery ward, found that after training and implementation, patients had higher morphine consumption [42]. In comparison, three studies showed no significant differences in medication across intervention and control groups [39, 43, 44]. No significant differences were found in additional treatment [43], arthritis referrals [39], or reducing doctor visits [39].

The effect PROMs have on tracking patient progress, evaluating and changing treatment is unclear. Surveys and interviews with clinicians identified mixed views, with additional conflicting results from trials testing the impact of PROMs on referrals and medication use.

### Potential implications for outcomes

Studies suggested that PROM use might influence patients’ health status, pain levels, and satisfaction. Two trials tested the impact of PROMs on patient outcomes, but no significant differences were found between the intervention and control groups on patient satisfaction [39, 45] or health status [39].

PROMs were also hypothesised to impact pain levels. Ravaud et al. [45] conducted a cluster-RCT; three wards were assigned to the intervention group and three wards assigned to control; the intervention group received education on pain and assessing pain with a visual analogue scale, and the scale was then used within the intervention wards. Pain significantly decreased in the intervention group compared to control (*d* = 0.1796 [0.0643–0.2949] *p* = −0.038) [45]. An additional study assessed whether pain assessment through PROMs changed clinical practice; case coordinators in the intervention group received training on PROMs and PROMs scores were put into a summary sheet for patients and clinicians, showed no significant differences between intervention and control groups for pain levels [44]. However, the intervention group did show some benefit in pain levels; they reported less pain related to strenuous activity at follow-up (*d* = 0.4253 [0.054–0.7966] *p* ≤ 0.05) [44].

There is no definitive evidence as to whether PROMs have an impact on health status, with only some studies showing significant differences. Studies showed no effect on patient satisfaction. Additionally, no studies examined adverse effects on patient outcomes.

## Discussion

Thirteen studies were identified and synthesised in order to explore the potential impact on the process and outcome of health care of implementing PROMs into routine clinical practice for non-malignant pain. Five areas of potential impact were identified and organised into three stages of treatment.

The synthesis indicated that PROMs may have some impact during the initial consultation process. Clinicians mostly believe the use of PROMs contributes in some way to the assessment of the patient with a purpose to understanding a patients’ pain [33–37, 39]. This finding corroborates a previous systematic review, which found that PROMs impacted the assessment of patients through acting as a screening tool and improving diagnosis [16].

PROMs were thought to affect the initial consultation through goal setting with the patient and decision-making for the course of treatment for a patient [33, 34, 36–38, 40]. This construct was assessed as high confidence because of moderate methodological limitations, with no concerns about coherence and adequacy. Another previous systematic review, examining qualitative literature on clinicians’ experiences of using PROMs, also identified that clinicians believed PROMs have potential to impact planning care and joint decision-making [17]. Whilst this review was not focused specifically on pain and examined more broadly the use of PROMs in clinical practice, these findings suggest that PROMs may have an impact on shared decision-making and treatment planning, not only in the treatment of non-malignant pain but also in other populations.

Results from qualitative literature identified that during the treatment process, clinicians and patients felt the use of PROMs had influence on the therapeutic relationship, through patient engagement and communication [33–37, 39]. This finding corroborates and extends the previous qualitative systematic review by Boyce et al. [17], finding that clinicians felt PROMs enhanced communication. A few quantitative studies contradicted these views, with surveys indicating that clinicians do not feel PROMs contribute to the therapeutic relationship or patient engagement [35, 39]. It is important to acknowledge that these results may be mutually compatible; although the results suggest that many clinicians feel PROMs influence the patient–clinician interaction and relationship, others may not have experienced this or feel this is the case. Further research is needed to explore why clinicians differ in their perceptions of PROMs; such work may help explain why PROMs do not always influence outcomes in trials.

There were also mixed findings on clinicians’ views about using PROMs to evaluate treatment and change treatment plans. Similarly, Greenhalgh and Meadows [15] discussed how only some clinicians within four included studies used the information from PROMs to change the treatment and care of their patients. Within our synthesis, many clinicians expressed that they used PROMs in this way [35, 37, 38, 40]; however, due to the lack of coherence and methodological limitations of the included studies, there is low confidence in this construct.

Using the qualitative literature from this synthesis to add the current knowledge in this area, it is important to note that some clinicians were concerned about the objectivity of data being provided [38]. Additionally, when un-validated PROMs are used their sensitivity to change and reliability are questionable, validated PROMs are essential if they are to track patient progress accurately, especially if results are being used to evaluate and change treatment plans.

Specific examples of modifying treatment discussed in the literature were changing medication and referrals to other clinicians. Despite a few clinicians believing that PROM data may aid medication decisions, there were conflicting results on medication use. Two studies reported small changes to medication use [41, 42], although other results were non-significant [39, 43, 44]. Results also suggested that although some clinicians felt the use of PROMs contributed to referrals [34, 39], it did not have any impact [39, 43]. A previous review also identified seven studies which indicated that PROM feedback to clinicians did not statistically increase referrals to clinicians and healthcare services; however, a further six studies did show a statistical increase [16]. These conflicting results indicate that there is currently a lack of understanding surrounding the full processes by which PROMs may influence referrals, and there may be additional variables that influence the referral process; further analysis should be undertaken to explore this area.

There is also conflicting evidence showing PROMs impact on patient outcomes. The results from this review showed limited to no improvement in pain levels and no significant improvement on patient satisfaction [39, 42, 44, 45]. Boyce and Browne [48] and Ravaud et al. [45] reviewed the usefulness of providing group-level feedback of PROMs to clinicians and included studies from various clinical practices and patient populations; patient populations that saw improvements were those with liver disease, and patients in mental health and oncology settings. These results may not be generalisable across study populations to include patients with non-malignant pain. Due to major concerns about the coherence of the data, substantial concerns over the richness of the data provided, and methodological limitations, there is very low confidence in this review construct. Although PROMs were hypothesised to impact pain levels, no studies investigated the impact on pain hypervigilance. If PROMs increase an awareness of pain and this is associated with pain catastrophising and hypervigilance, this could stimulate avoidance behaviours which may negatively impact patients’ health-related quality of life [46]. This is an area for future research.

This review synthesised a diverse body of evidence in accordance with CIS methodology. This generated an understanding of the complexity of PROMs, incorporating multiple perspectives. Due to the heterogeneity of the study designs, and small sample of papers, it is impossible to run sub-group analyses. For example, not all studies detailed whether patients had acute or chronic pain, two studies included both medical and surgical patients, and some studies employed a mix of validated and non-validated PROMs.

Previous research has been conducted to assess which style of PROM is the most precise to measure clinical pain intensity [47]. It should be acknowledged that within clinical practice, clinicians may use the tool they deem the most relevant and appropriate for specific patients, as well as considering validation [34]. Therefore, studies using non-validated PROMs were included in this review to reflect the use of PROMs in clinical practice. As there is no current literature on the most effective method to implement and use PROMs in clinical practice for non-malignant pain, all measures, populations, settings, and perspectives were eligible for review. Finally, barriers to successful implementation, such as clinician knowledge and education, organisation support, selection of outcome measure, and application of PROMs, were deemed beyond the scope of the review [26]. However, these are important issues which need to be addressed in future research to evaluate the impact of PROM use.

## Conclusion

The synthesis provided preliminary evidence to suggest that PROMs may be having some impact and that some clinicians and patients believe they could be useful in the treatment of pain. PROMs potentially impact clinical practice throughout the treatment process, through assessment of patients, decision-making, therapeutic relationship, tracking progress and evaluating and changing treatment, and potential implications for outcomes. As there is currently a lack of clear evidence from the literature, it is premature to make definitive recommendations for how PROMs could be used in non-malignant pain. All of the constructs emerging from the synthesis would benefit from more exploration and further focused research. Further pre-clinical research needs to develop the theoretical basis for PROM use in treatment of non-malignant pain, to describe and predict how PROMs work. A better understanding of potential effects and mechanisms will aid the generation of hypotheses to evaluate more effectively the role of PROMs in clinical practice for non-malignant pain. Future research should evaluate the clinical and psychosocial consequences of using PROMs and associated mechanisms, through randomised controlled trials and process evaluations.
